# Antiviral and virucidal activities of *Duabanga grandiflora* leaf extract against Pseudorabies virus in vitro

**DOI:** 10.1186/s12906-016-1120-2

**Published:** 2016-05-23

**Authors:** Farhah Zaidar Abdul Malik, Zeenathul Nazariah Allaudin, Hwei San Loh, Ting Kang Nee, Homayoun Hani, Rasedee Abdullah

**Affiliations:** Department of Veterinary Pathology and Microbiology, Faculty of Veterinary Medicine, Universiti Putra Malaysia, Serdang, 43400 Selangor Malaysia; School of Biosciences, Faculty of Science, University of Nottingham Malaysia Campus, Jalan Broga, 43500 Semenyih, Selangor Malaysia; Biotechnology Research Centre, The University of Nottingham Malaysia Campus, Semenyih, Selangor Malaysia; Department of Biomedical Sciences, Faculty of Science, The University of Nottingham Malaysia Campus, Semenyih, Selangor Malaysia; Department of Veterinary Laboratory Diagnostics, Faculty of Veterinary Medicine, Universiti Putra Malaysia, Serdang, 43400 Selangor Malaysia

**Keywords:** Antiviral assay, *Duabanga grandiflora*, Plaque reduction assay, Inhibition assay, Virucidal assay

## Abstract

**Background:**

*Duabanga grandiflora* or known in Malaysia as *Berembang Bukit*, *Megawasih*, or *Pedada Bukit*, is a native plant of the Southeast Asian countries. In this study, the anti-viral properties of *D. grandiflora* were investigated.

**Methods:**

The *D. grandiflora* leaf extracts were obtained with ethyl acetate, hexane, and ethanol as solvents and labelled 37 leaf ethyl acetate (37 L EA), 37 leaf hexane (37 L H), 37 leaf ethanol (37 L ET), respectively. The cytotoxicity of the extracts on Vero cells were determined by the 3-(4,5-Diamethylthiazol-2-yl)-2,5-Diphenyltetrazolium Bromide (MTT) assay.

**Results:**

Among extracts, 37 L EA was most cytotoxic to Vero cells, followed by 37 L H and 37 L ET, with CC_50_ of 218, 833, and >1000 μg/mL, respectively. The cytopathic effect (CPE) and plaque reduction, inhibition, and virucidal assays and the selective index (SI) were employed to determine the effect of the extracts on infectivity and replication of pseudorabies virus (PrV) in Vero cells. The *D. grandiflora* leaf extracts showed dose-dependent antiviral activities, with higher activities at high doses. The 37 L ET and 37 L EA showed anti-viral effects through plaque formation and viral replication inhibitions, and virucidal property. The SI of the 37 L ET and 37 L EA by the viral replication inhibition assay was 8.3 and 1.9, respectively, and by the CPE reduction assay, 6.7 and 2.9, respectively.

**Conclusion:**

Ethanol is the best solvent for the preparation of *D. grandiflora* leaf extract as an antiviral agent.

**Electronic supplementary material:**

The online version of this article (doi:10.1186/s12906-016-1120-2) contains supplementary material, which is available to authorized users.

## Background

*Duabanga*, a genus originally included in the *Sonneratiaceae*, is now classified under the subfamily Duabangoideae of the family *Lythraceae. Duabanga*, native to Southeast Asia, consists of three species, *Duabanga grandiflora*, *D. moluccana*, and *D. taylorii Jayaweera* [1]. In Malaysia, *Duabanga* is known as *Berembang Bukit*, *Megawasih* and *Pedada Bukit. Duabanga grandiflora* contains eugeniin, comprising of gallic acid, ellagic acid, and sugar [2].

Traditionally, the *Duabanga grandiflora* tree has several medicinal properties. The leaf extract is used topically to whiten skin, retard aging, and heal inflammation through the stimulation of type II collagen production [2]. The *D. grandiflora* extracts and partial purified fractions exhibited bacterial growth inhibition and re-sensitized methicillin-resistant *Staphylococcus aureus* (MRSA) towards ampicillin treatment by inhibiting MRSA biofilm formation and the penicillin-binding protein, PBP2a [3–6]. The plant extracts also possess insecticidal activity [7].

The antimicrobial and phytochemical properties of *D. grandiflora* was first described in 2011 [3] in a report that described the use of higher plant species for combating newly emerging and drug resistant pathogens. The ethanol *D. grandiflora* leaf extract appears to contain a high concentration of phytochemicals including tannins, phenolic compounds, flavonoids and steroids. On the other hand, the ethyl acetate *D. grandiflora* extract contain high level of tannins and moderate levels of alkaloids and phenols [3]. Previously, phytochemicals especially tannins, phenolic compounds, flavonoids were reported to possess antiviral activities against human and animal viruses [8–12].

Herpesviruses are highly prevalent in humans and most animals [13]. Members of Herpesviridae are host-specific agents that share properties, which allow them to cross species barriers [14, 15]. This group of viruses has relatively short reproduction cycle, can efficiently destroy infected cells, spread rapidly, and establish latent infection in the host.

In this study, the antiviral properties of *D. grandiflora* plant extracts against PrV was determined. The cellular toxicity and the most effective solvent to be used in the preparation of *D. grandiflora* leaf extracts were also determined.

## Methods

### Crude plant extract

*D. grandiflora* was collected from the Semenyih Dam area (3.079066°N 101.886091°E), Selangor, Malaysia, identified by Dr. Christopher Wiart of the University of Nottingham, Malaysia Campus, and the herbarium voucher specimens (UNMC 37) deposited at the Herbarium of Faculty of Science of the University. Crude plant extracts, harvested from the leaves, extracted with hexane, ethanol, and ethyl acetate, and labelled 37 L H, 37 L ET, and 37 L EA, respectively were prepared by Dr. Kang Nee Ting and Prof. Dr. Hwei San Loh of the University of Nottingham, Malaysia.

The leaves of the *D. grandiflora* were cleaned with sterile deionized water, cut into small fragments, and dried in a closed room at 25 to 28 °C for 2 weeks. The dried leave fragments were grounded, weighed and soaked in 95 % ethanol at a proportion of 1:8 (fragment: ethanol) at room temperature for 24 h. The plant materials were then subjected to sequential fractionation using hexane, ethanol, and ethyl acetate. Each plant material was soaked and fractionated trice before subjecting to rotary evaporation at 40 °C under reduced pressure. The concentrated crude extracts were stored at −20 °C.

### Pseudorabies Virus (PrV) propagation

The PrV strain AIP used in this study is from the working stock virus (1 × 10^8^ pfu/mL) prepared and stored at - 80 °C in the Virology Laboratory, Faculty of Veterinary Medicine, Universiti Putra Malaysia. Quantitation of stock virus was by plaque titration assay using Vero cells.

### Vero cell culture

Vero cells (ATCC No. CCL-81) were grown in RPMI 1640 supplemented with 10 % fetal bovine serum (FBS) and antibiotic-antimycotic combination [1 % Penicillin (100 U/mL), 1 % Streptomycin (100 mg/mL) and 1 % Fungi zone (2.5 mg/mL)]. The cells were maintained in RPMI maintenance media supplemented with 1 % FBS, seeded into sterile 96-well flat bottom and 24-well plates at 1 × 10^4^ cells/well, and incubated under 5 % CO_2_ humidified atmosphere at 37 °C.

### Plant extract

All plant extracts were dissolved, with frequent shaking for 48 h in dimethyl sulfoxide (DMSO) and then diluted with sterile deionized water to obtain stock concentrations of 10 mg/mL in 10 % DMSO and 50 mg/mL in 50 % DMSO. The 37 L ET extract was the most soluble followed in order by 37 L EA, and 37 L H. The solutions were stored as stock at −20 °C.

### Cytotoxicity assay

The stock solutions were diluted serially with RPMI media without FBS to obtain extract concentrations of 62.5, 125, 250, 500, and 1000 μg/mL. In general, the final concentration of DMSO in the working concentration was below 0.3 %. 100 μL of each concentration was used to treat confluent monolayer Vero cells in a 96-well flat-bottom plate. For reference the Vero cells were treated with 100 μL of either 0.05, 1.0, 10, or 12 % DMSO in PBS. Non-treated Vero cells served as the negative control. Positive controls were treated with DMSO at the same concentrations as the plant extracts. All experiments were performed in triplicates. The Vero cells were then incubated for 48 h under 5 % CO_2_, humidified atmosphere at 37 °C and visualized under light microscopy. The cytotoxic effect of treatments was determined by MTT assay. Cytotoxicity scoring was according a 5-point scale: 5 = confluent well-defined monolayer with cell-to-cell contact and the cell morphology and density intact; 4 = occasional cell lysis with ≤20 % of the cells round in shape, loosely attached, devoid of intracytoplasmic granule; 3 = prevalent cell lysis with <50 % of cells round in shape and devoid of intracytoplasmic granule; 2 = the majority of the cells are affected but <70 % of cells are round in shape or lysed; 1 = almost total lysis and destruction of cells and presence of spaces between cells [16]. The cytotoxic concentrations (CC_50_) of crude plant extracts, which are the concentrations causing 50 % cell cytotoxicity of Vero cells were determined (Additional file 1).

### Antiviral assays

#### Cytopathic Effect (CPE) reduction assay

The CPE reduction assay was conducted according to method described previously [17]. The Vero cells were seeded in a 96-well flat-bottom plate for 24 h to obtain a monolayer. 50 μL of 50 TCID_50_ PrV with 50 μL of either 18.75, 37.5, 75, 150, or 300 μg/mL extracts was added to the cells and incubated under 5 % CO_2_ at 37 °C for 72 h. The CPE was determined under light microscopy and graded as follows: 0 = 0 %, 1 = 0 to 25 %, 2 = 26 to 50 %, 3 = 51 to 75 %, and 4 = 76 to 100 % CPE. The percentage CPE inhibition was calculated using the following formula: % inhibition = (CPE_exp_/CPE_control_) × 100, to determine the 50 % effect concentration (EC_50_) of the extracts where CPE_exp_ = CPE of extract and CPE_control_ = CPE of virus control.

### Plaque reduction assay

Plaque reduction assay was conducted according to the method described by Hsuan et al. [18]. Briefly, confluent monolayers of Vero cells were grown in 24-well plates. 100 μL of extracts at either 62.5, 125, 250, 500 μg/mL or 100 μL of 100pfu PrV were added to the monolayer Vero cells and incubated for 90 min under 5 % CO_2_ at 37 °C. Cells treated virus but not with plant extract served as the positive control while those not treated with either virus or extract served as the negative control. The incubating solution contain virus and extract was discarded and 1 mL 1 % methylcellulose in DMEM containing 2 % FBS added to the cells and the plates incubated under 5 % CO_2_ for 48 h at 37 °C. The infected cells were fixed with 10 % formal saline, stained with 0.5 % crystal violet solution, and the plaques counted. The 50 % plague inhibition concentration (IC_50_) was determined by the following formula [19]: % inhibition = [1 – (pfu_exp_/pfu_control_)] × 100, where pfu_exp_ = plague formation by extract-and virus-treated cells and pfu_control_ = plague formation by virus-treated cells.

### Inhibition assay on viral replication

The viral replication inhibition assay was according to that described previously [20]. 100 μL of 100pfu PrV were incubated with the monolayer Vero cells in a 24-well flat-bottom plate under 5 % CO_2_ at 37 °C for 90 min. The incubating solution was discarded, the cells treated with 0.5 mL of either 25, 50, 100, or 200 μg/mL extract in 1 % methylcellulose and reincubated under 5 % CO_2_ at 37 °C for 48 h. The infected cells were fixed with 10 % formal saline and stained with 0.5 % crystal violet solution. The plaques were counted and the IC_50_ calculated as described in plaque reduction assay.

### Determination of extracellular virucidal assay

The extracellular virucidal activity of extracts on PrV was performed according to the method described by Carlucci et al. [21]. Monolayer Vero cells were treated with 5 × 10^6^ pfu PrV and 100 μL of either 62.5, 125, 250, or 500 μg/mL extract and incubated at 25 °C for 6 h, followed by 10-fold serial dilution. 100 μL mixture of equal amounts of virus (50 μL of 1 × 10^8^/mL) and extract (50 μL) in 2 % FBS/DMEM was incubated under 5 % CO_2_ for 90 min at 37 °C. The incubating solution containing virus and extract was discarded, 0.5 mL of 1 % methylcellulose in 2 % FBS/DMEM was added to the cells and the plate incubated under 5 % CO_2_ at 37 °C for 48 h. The plaques formed were fixed with 10 % formal saline and stained with 0.5 % crystal violet solution for 30 min and counted. Residual virus infectivity was determined according the following formula: plague formation (pfu) = Number of plaques × [1/viral inoculation] × [1/dilution fold]. The IC_50_ of extracts of plaque inhibition was calculated as follows: IC_50_ = (pfu_exp_/pfu_control_) × 100, where pfu_exp_ = plague formation by extract- and virus-treated cells and pfu_control_ = plague formation by virus-treated cells.

### Data analysis

All values are expressed as mean ± standard deviation. The CC_50_, EC_50_, and IC_50_ values were estimated by extrapolating the graph plotted with software Microsoft Excel 2007. Mean values were compared using multiple comparison Tukey test with the SPSS 6.0 software. The difference was statistically significant when *p* < 0.05.

## Results

### Cytotoxicity assay

**Fig. 1:**
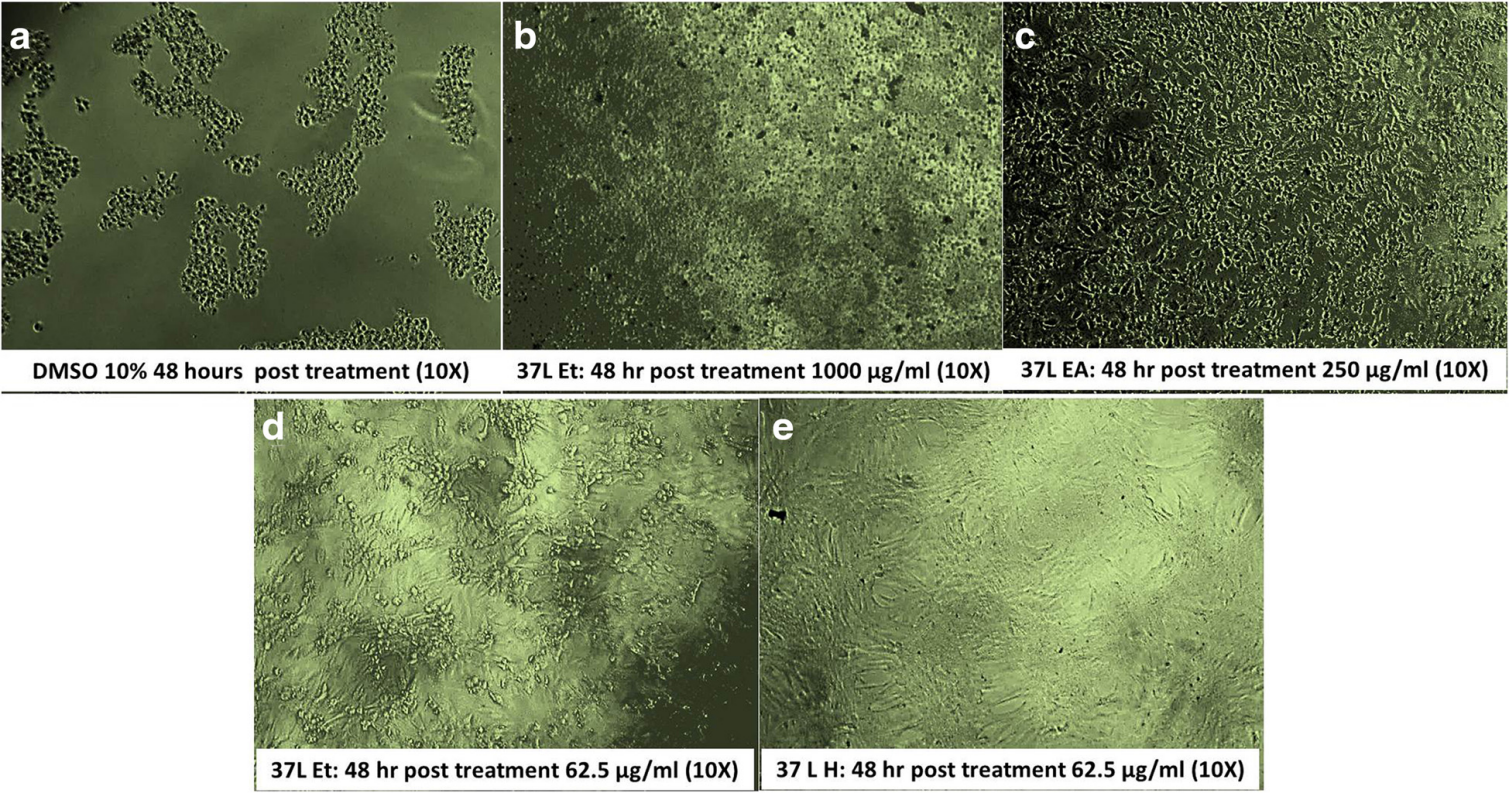
Cytotoxicity of Vero cells to *D. grandiflora* leaf extract. **a** Representative of score 1 cytotoxicity showing almost complete cell destruction and lysis of the cells (~100 % of cells were lysed); **b** representative of score 2 cytotoxicity where the majority of the cells appear rounded and lysed. The black dots are pyknotic cells (~70 % of cells were lysed); **c** representative of score 3 cytotoxicity with prevalent cell lysis. The cells appear rounded and devoid of intracytoplasmic granules (~50 % of cells were rounded); **d** representative of score 4 cytotoxicity with occasional cell lyses. Approximately 20 % of cells appear rounded, loosely attached, without intracytoplasmic granules; **e** representative of score 5 cytotoxicity showing confluent monolayer of well-defined cells with cell-to-cell contact (~100 % of culture flask surface were covered by cells). The cell morphology and density were not affected by extract treatment

**Fig. 2:**
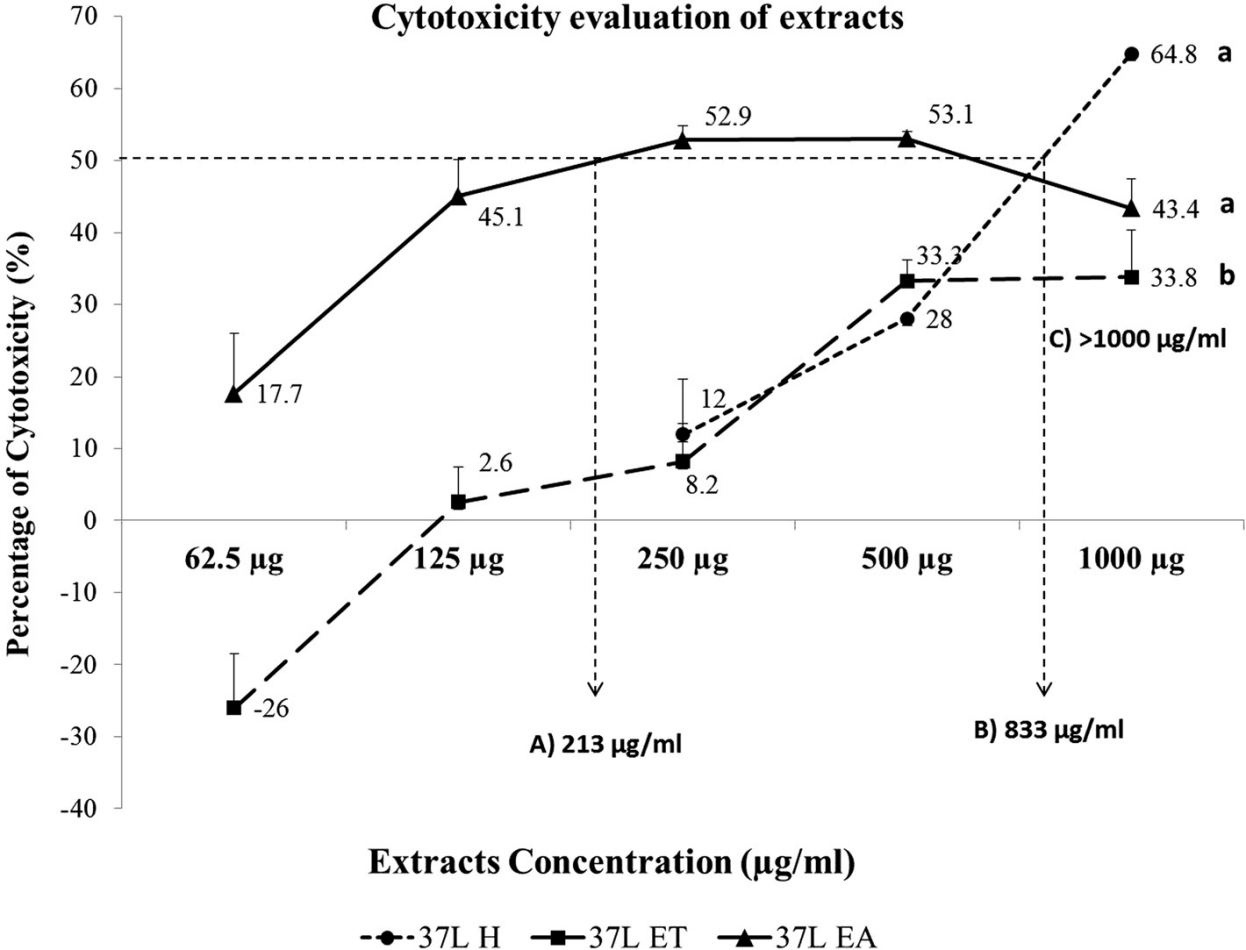
Determination of IC_50_ of *D. grandiflora* leaf extract on Vero cells. The CC_50_ of Vero cell treated with 37 L EA, 37 L H, and 37 L ET was 213, 833, and >1000 μg/mL, respectively. “a and b” letter indicate significant difference between individual extracts at *p* < 0.05

The CC_50_ of 37 L EA and 37 L H on Vero cells was 213 and 813 μg/mL, respectively. For 37 L ET, the CC_50_ was >1000 μg/mL with <50 % cytotoxicity (Table 1, Figs. 1 and 2). The 37 L H and 37 L EA extracts were significantly (*p* < 0.05) more cytotoxic than 37 L ET extract to Vero cells.

**Table 1:** Cytotoxicity scoring of

Conc. (μg/mL)	*D. grandiflora* leaf extract		
	37 L H	37 L ET	37 L EA
62.5	3	3	3
125	3	3	3
250	3	2	2^a^
500	2	2	2
1000	2^a^	2^a^	2

^a^Scored for concentration near to CC_50._ Score 1 = severely cytotoxic. Almost complete cell destruction and lysis of the cells (~100 % of cells were lysed), 2 = significantly cytotoxic (~70 % of cells were lysed), 3 = moderately cytotoxic (~50 % of cells were rounded), 4 = mildly cytotoxic. Approximately 20 % of cells appear rounded, loosely attached, without intracytoplasmic granules, 5 = non-cytotoxic (~100 % of culture flask surface were covered by cells). 37 L H, 37 L ET, and 37 L hexane, ethanol, and ethyl acetate extract, respectively

### Antiviral assay

#### CPE reduction assay

**Fig. 3:**
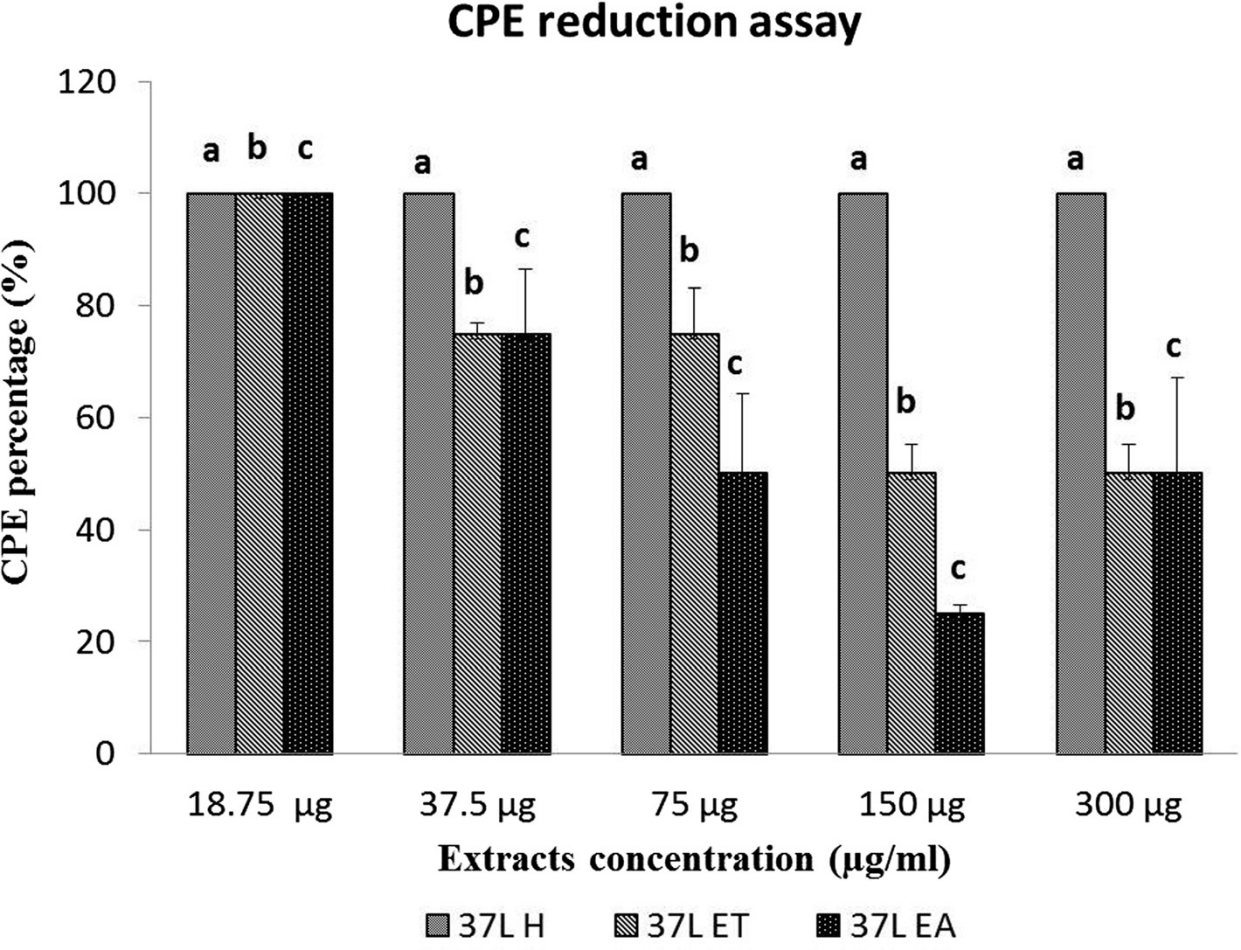
Effect of *D. grandiflora* leaf extracts on PrV-induced cytopathic effect on Vero cells. a,b,c indicate significant difference between individual extracts in each concentration at *p* < 0.05

The anti-PrV activity of *D. grandiflora* leaf extracts were evaluated by the CPE reduction assay after 72 h of incubation. The 37 EA and 37 ET extracts showed a dose-dependent effect in PrV-induced CPE on Vero cells with EC_50_ of 75 and 150 μg/mL, respectively (Fig. 3). The 37 L H extract did not show the same effect.

### Plaque reduction assay

**Fig. 4:**
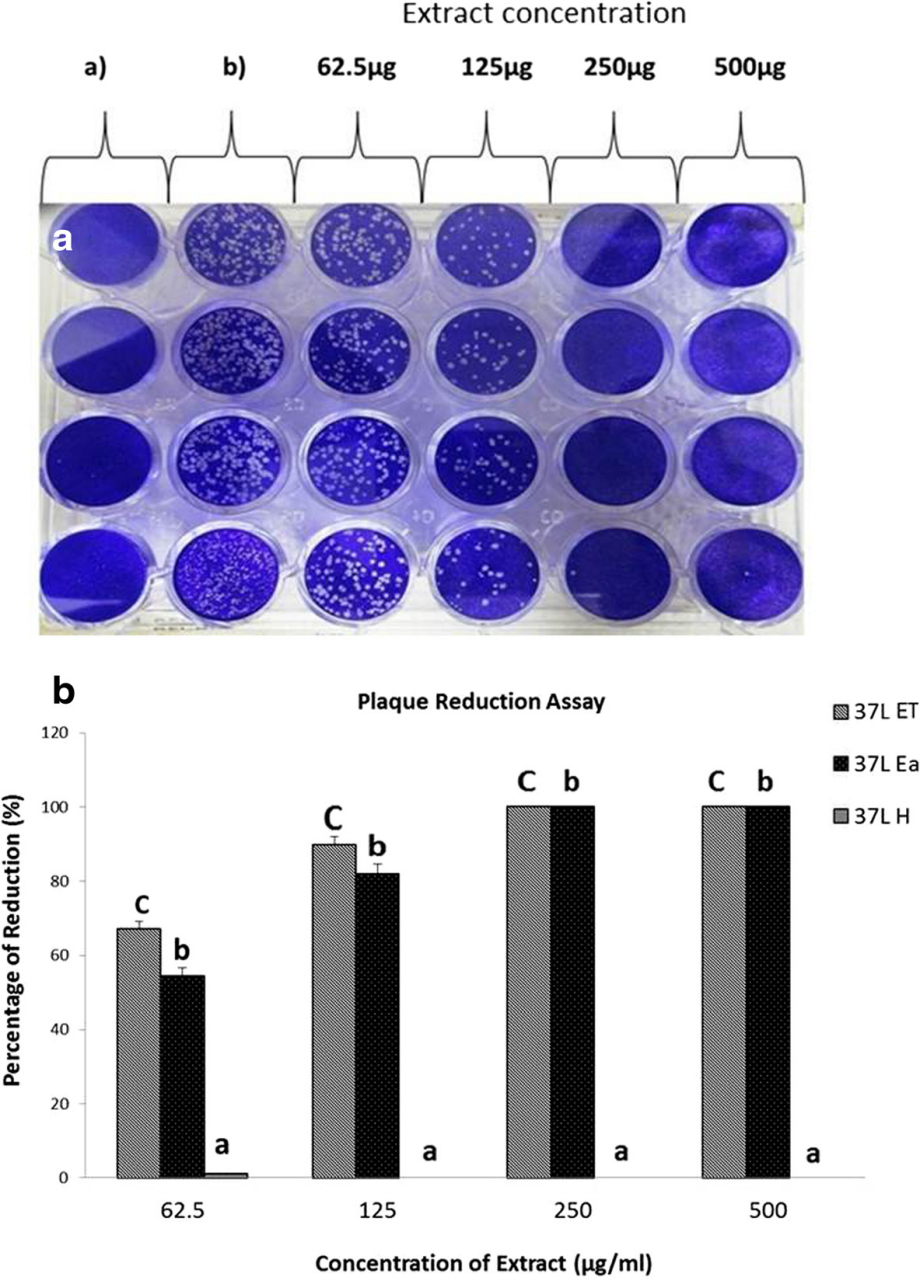
Plaque reduction assay of Vero cells infected with PrV pre-treated with *D. grandiflora* leaf extracts. (A) Plates show reduction in plaque formation; a) Negative control, not treated. b) Positive control treated with PrV only. Greater reduction in plague formation with increase in extract concentration. No plaque formation at 250 and 500 μg/mL extract. (B) Quantification of plague reduction. a,b,c indicate significant difference between individual extracts in each concentration at *p* < 0.05

In the plaque reduction assay, the Vero cells were infected with 100 pfu of PrV and 100 μL of either 62.5, 125, 250, or 500 μg/mL plant extract. Extracts 37 L ET and 37 L EA caused reduction in plaque formation with 37 L ET and 37 L EA causing 100 % reduction at 250 and 500 μg/mL, respectively (Fig. 4). However, 37 L H did not produce any effect on the Vero cells.

### Viral replication inhibition assay

**Fig. 5:**
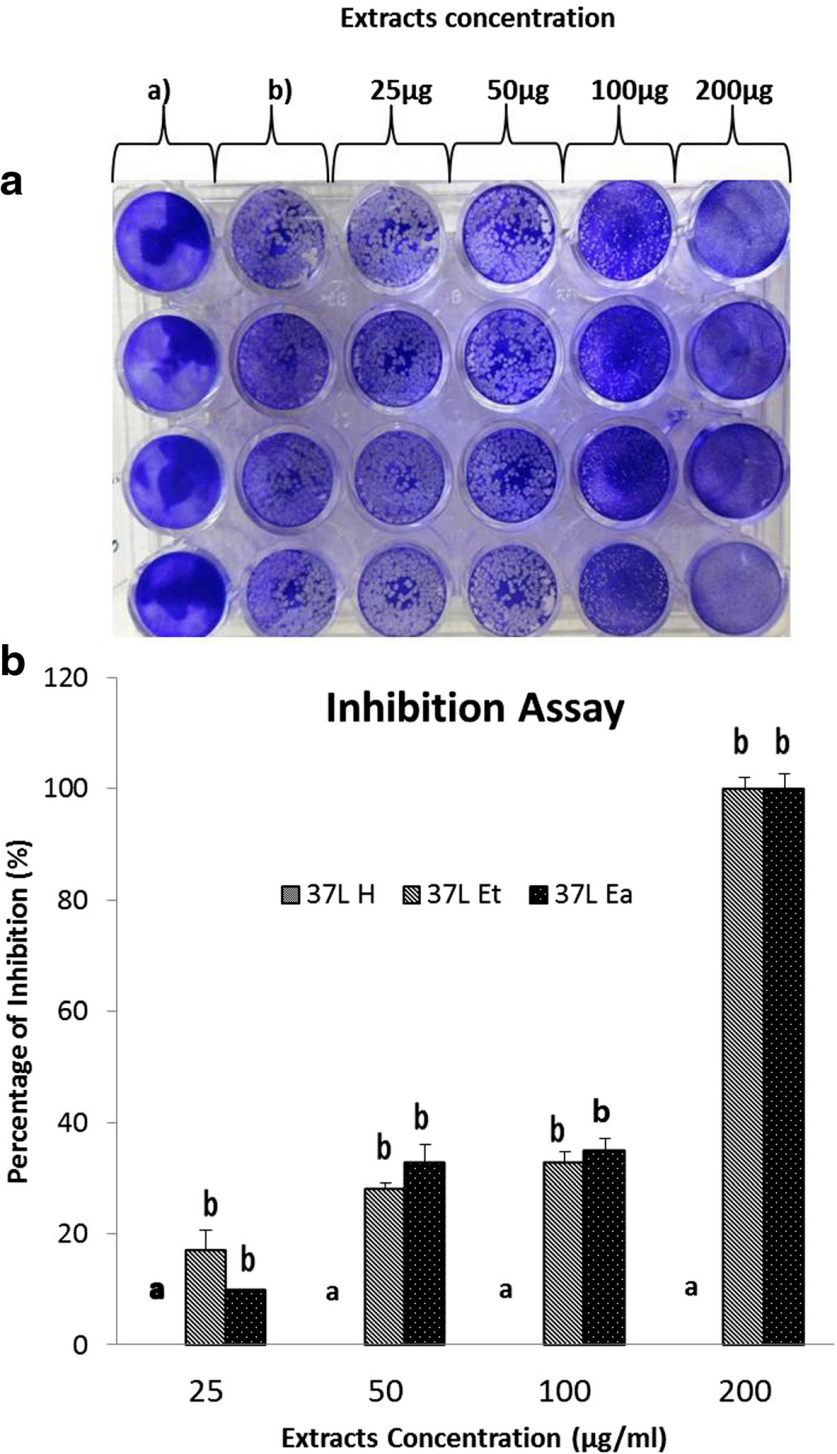
Inhibition of viral replication in PrV-infected Vero cells after treatment with *D. grandiflora* leaf extract. **a** Plates show reduction in plaque numbers in extract-treated PrV-infected cells; a) Negative control, untreated, without plaque formation, b) Positive control, PrV-treated only. The number of plaque decreased with increase in extract concentration. **b** Quantification of plague inhibition by *D. grandiflora* leaf extract by difference solvents on PrV replication. a,b indicate significant difference between individual extracts in each concentration at *p* < 0.05

The Vero cells were infected with PrV for 90 min and then treated with either 25, 50, 100, or 200 μg/mL *D. grandiflora* leaf extracts to determine their effect on virus replication. The results showed that 37 L H did not inhibit plaque formation. However, 200 μg/mL 37 L Et and 37 L EA caused complete inhibition in plaque formation indicating total inhibition of viral replication in Vero cells (Fig. 5).

### Virucidal activity

**Fig. 6:**
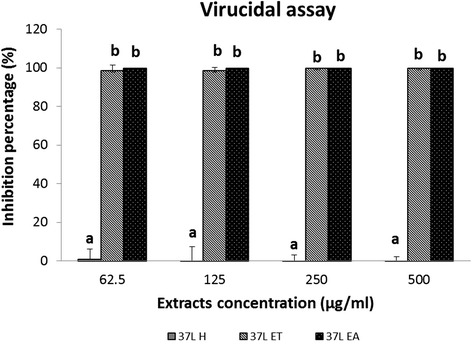
Virucidal effect of *D. grandiflora* leaf extracts. a, b indicate significant difference between individual extracts in each concentration at *p* < 0.05

*D. grandiflora* leaf extracts were incubated with PrV to determine their effect on the virus and its residual infectivity. The results showed that 37 L EA at 62.5 μg/mL had no residual viral infectivity in the Vero cells indicating total viral inactivation. The 37 L ET only had slight residual infectivity of 1.3 % that is translated as 98.7 % viral inactivation. On the other hand, 37 L H produced 100 % residual infectivity indicating no inhibitory effect on the viral replication in Vero cells (Fig. 6).

The selective index (SI) [CC_50_/IC_50_] was estimated by the viral inhibition and CPE assays. Based on the SI, 37 L ET extract has the highest antiviral potential while 37 L H extract had the lowest.

**Fig. 7:**
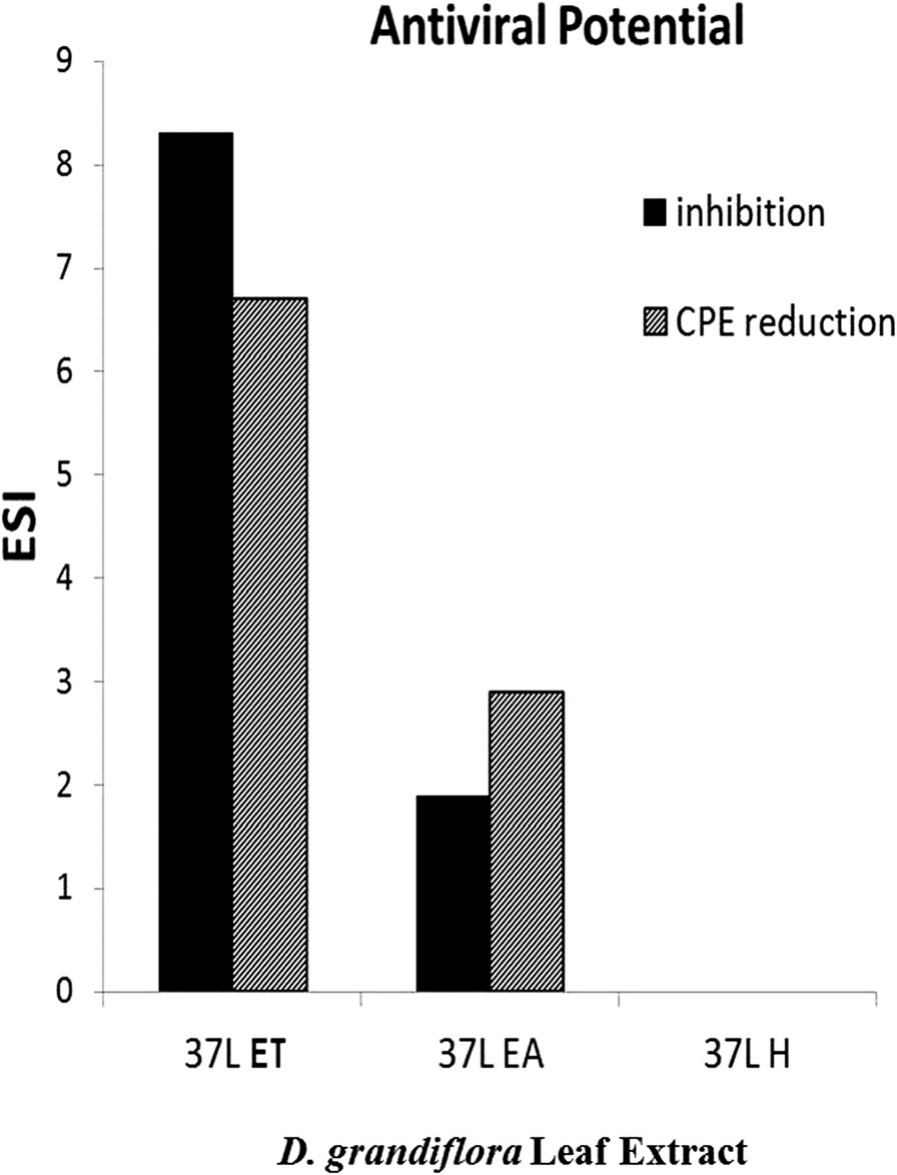
Estimated selective index (ESI) of *D. grandiflora* leaf extract. Ethanol extraction of *D. grandiflora* leaves produced compounds with the highest antiviral potential

These results showed that ethanol is the best solvent for the extraction of compounds with virucidal properties from *D. grandiflora* leaves (Fig. 7).

## Discussion

The study showed that the cytotoxicity of *D. grandiflora* leaf extracts increased with concentration and this is attributed to their bioactive compound content. It was earlier shown that chloroform *D. grandiflora* extract contains 3 triterpenes, 3 flavones, 4 phenolic compounds and 1 steroids [22]. Some of these compounds and their derivatives possess antimicrobial properties [7]. In this study, we determined the antiviral activity of the *D. grandiflora* leaf extract obtained by using hexane, ethanol, and ethyl acetate as solvents. It appears that the ethanol *D. grandiflora* leaf extract had the highest antiviral activity while being less toxic to Vero cells than either the hexane or ethanol extract.

DMSO usage as diluent of extracts at concentrations of less 10 % is not cytotoxic [23]. Our study showed that 0.1 % DMSO produced approximately 3 % Vero cell toxicity. Thus, at this concentration, DMSO did not contribute significantly to the cytotoxic effect of *D. grandiflora* leaf extract.

Many plants extracts have been shown to inhibit or inactivate virus replication. Among extracts with antiviral properties is the *Isatis indigotica* extract that produced a dose-dependent inhibition of PrV replication in vitro [18] and the *Geranium sanguineum* extracts that inhibit herpesvirus infection by causing perturbations in the early stages of virus replication [17]. *D. grandiflora* leaf extracts seems to have similar antiviral potentials. It was shown in this study that the extract inhibits viral infection via the cytopathic effect, plaque reduction, viral replication, and virucidal activities. However, the antiviral activities of the *D. grandiflora* leaf extracts are dependent on the solvent used in the preparation of the extract. The hexane extract did not have any inhibitory activity towards PrV. Based on the CPE, virus inhibition and virucidal assays, the ethanol and ethyl acetate *D. grandiflora* leaf extracts have equal virucidal and inactivation activity before the PrV could infect Vero cells [24]. The CPE of the extracts is dose-dependent with the most effective antiviral concentrations assumed to be from 150 to <300 μg/mL. Increasing the extraction concentration to 300 μg/mL did not seem to improve the antiviral effect of either the ethanol or ethyl acetate *D. grandiflora* leaf extract.

The *D. grandiflora* leaf extracts seemed to inhibit PrV infection of Vero cells. This is clearly evident by the plaque reduction assay where extract treatment prevented Vero cell plague formation, particular at concentrations of ≥250 μg/mL. In fact, all *D. grandiflora* leaf extracts prevented PrV infection of Vero cells. However, it should be noted that in in vitro experiments, viral infection of cells is dependent on factors such as virus concentration and incubation period for viral infection. In this study, the virus concentrations and incubation periods for CPE and plague reduction assays were variable. In the CPE assay, although the incubation period was longer, less virus was used than in the plague reduction assay. For that reason, the results from the two assays were not completely complimentary. There was total inhibition of plague formation in infected Vero cells treated with high concentrations of ethanol and ethyl acetate *D. grandiflora* leaf extracts while the cytopathic effect was between 20 and 50 %.

In the evaluation of virucidal activity, the PrV were pre-incubated with *D. grandiflora* leaf extracts for 6 h before infecting Vero cells. The antiviral effect of the extracts was determined by the viral residual infectivity in Vero cells. The ethanol *D. grandiflora* leaf extract at all concentrations used in the study almost completely inhibited plague formation. To confirm the antiviral potential of the extracts, the SI was calculated. A high SI suggests that the extract would has good antiviral properties. In this study, the ethanol had at least twice as high SI than the ethyl acetate *D. grandiflora* leaf extract. Based on these results, the ethanol *D. grandiflora* leaf extract is most appropriate to be developed into a therapeutic compound for viral diseases.

The efficacy of anti-viral drugs can be determined by their IC_50_ on Vero cells. The anti-herpesvirus drug, Acyclovir for example, has an IC_50_ of 0.8 to 0.9 μg/mL on Vero cells [25, 26]. The ethanol and ethyl acetate *D. grandiflora* leaf extracts was shown to have much lower IC_50_ than Acyclovir at 0.12 and 0.112 μg/mL, respectively suggesting that the *D. grandiflora* leaf extracts are more efficacious in the treatment of herpesvirus infections than Acyclovir.

The antiviral activities of the ethanol and ethyl acetate *D. grandiflora* extracts are attributed to their phytochemical contents [3], particularly tannins, phenolic compounds, flavonoids, and alkaloids that may act synergistically. Among the advantages of crude extracts as antiviral agents are their greater potency, fewer side effects and toxicity, and cost-effectiveness [27–29]. Although, further studies would be of interest to determine whether the components of the *D. grandiflora* leaf extracts have synergistic effects, this does not detract from fact that the extracts have effective anti-herpesvirus properties.

## Conclusion

The ethanol *D. grandiflora* leaf extracts while showing the least cytotoxic effect towardsVero cells, is the most efficacious anti-viral agent among the *D. grandiflora* leaf extracts investigated in this study. The wide safety margin, low cytotoxicity and high selective index make the ethanol *D. grandiflora* leaf extract a prime candidate for development as an anti-viral agent.

## Additional file

Additional file 1: Supplementary document DMSO Cytotoxicity assay. (JPG 40 kb)

## Abbreviations

ATCC: American Type Culture Collection
CC_50_: the cytotoxic concentrations
CPE: cytopathic effect
DMEM: Dulbecco modified Eagle medium
DMSO: dimethyl sulfoxide
EA: ethyl acetate
EC_50_: 50 % effect concentration
ET: ethanol
FBS: fetal bovine serum
H: hexane
IC_50_: 50 % inhibitory concentration
MRSA: methicillin-resistant *Staphylococcus aureus*
MTT: 3-(4,5-Diamethylthiazol-2-yl)-2,5-Diphenyltetrazolium Bromide
PBP2a: penicillin-binding protein
PFU: plaque forming unit
PrV: Pseudorabies virus
RPMI: Roswell Park Memorial Institute
SI: selective index
TCID_50_: 50 % tissue culture infective dose
