# Supplementary figures and images for: Antiviral and virucidal activities of Duabanga grandiflora leaf extract against Pseudorabies virus in vitro
Source: BMC Complement Altern Med. 2016 May 23;16:139. doi: 10.1186/s12906-016-1120-2 (PMC4877979; doi:10.1186/s12906-016-1120-2)

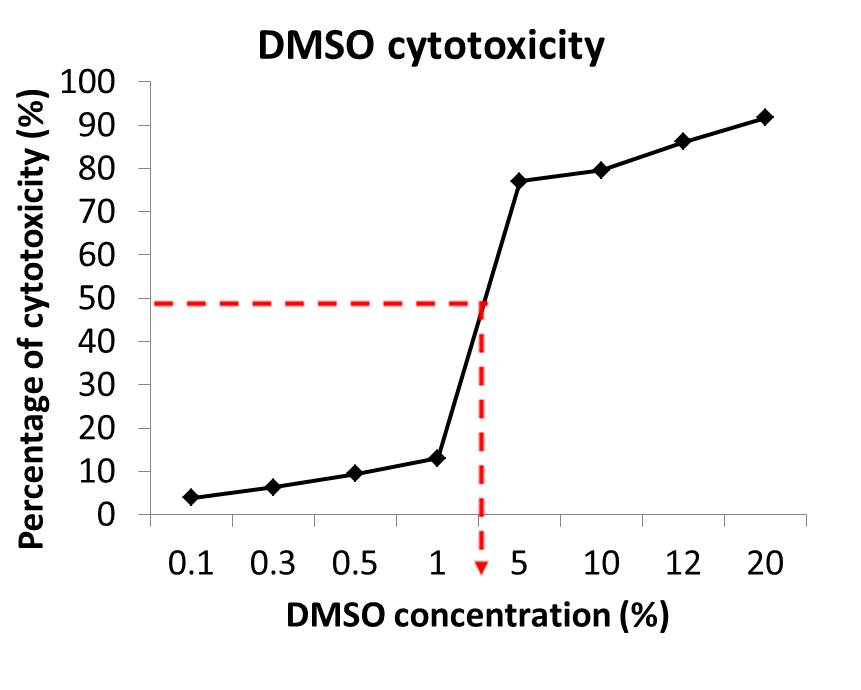

Supplement: Additional file 1: — Supplementary document DMSO Cytotoxicity assay. (JPG 40 kb) [file 12906_2016_1120_MOESM1_ESM.jpg]
